# Aerobic Exercise for Cognitive and Functional Enhancement in Schizophrenia: Evidence, Mechanisms, and Practical Recommendations

**DOI:** 10.1093/schizbullopen/sgag006

**Published:** 2026-03-21

**Authors:** Joseph Ventura, Lukas Roell, Isabel Maurus, Joseph Firth

**Affiliations:** UCLA Department of Psychiatry, Semel Institute for Neuroscience and Human Behavior, 300 Medical Plaza, Room 2243, Los Angeles, CA 90095, USA; Department of Psychiatry and Psychotherapy, LMU University Hospital, NeuroImaging Core Unit Kraepelinstrasse 10, 80804, Munich (NICUM) Germany; LMU University Hospital, Max Planck Institute of Psychiatry, Kraepelinstrasse 10, 80804, Munich, Germany; Department of Psychiatry and Psychotherapy, LMU University Hospital, NeuroImaging Core Unit Kraepelinstrasse 10, 80804, Munich (NICUM) Germany; Division of Psychology and Mental Health, University of Manchester, Manchester Academic Health Science Centre, Oxford Road, Manchester, M13 9PL, United Kingdom; Greater Manchester Mental Health NHS Foundation Trust, Manchester Academic Health Science Centre, Oxford Road, Manchester, M13 9PL, United Kingdom

**Keywords:** aerobic exercise, neurocognition, daily functioning, exercise training recommendations, first episode psychosis, quality of life, psychiatric symptoms, multi-episode schizophrenia

## Abstract

Schizophrenia is a psychiatric condition often characterized by cognitive deficits which precede the onset of positive symptoms and contribute significantly to enduring functional impairments and poor quality of life. Those cognitive deficits can have a profoundly negative impact on daily activities such as social relationships and work functioning. Although pharmacological interventions remain central to the treatment of schizophrenia (SZ), they have a negligible impact on cognitive functioning. Yet multiple reviews and meta-analyses support the hypothesis that Aerobic Exercise (AE) could serve as an adjunctive, therapeutic intervention for these cognitive impairments. AE is recommended for individuals with Multi-episode Psychosis (MEP) and with First Episode Psychosis (FEP). This body of evidence indicates that for individuals with SZ, engagement in AE shows measurable enhancements in specific brain regions associated with key domains of neurocognitive functioning. AE has also been associated with improvements in neurobiological processes including increased BDNF, enhanced neurogenesis, and improved neural connectivity. These improvements were linked to key cognitive domains associated daily functioning such as attention, working memory, reasoning, speed of processing, and social cognition. Engagement in AE has also been associated with improvements in Activities of Daily Living, reductions in psychiatric symptoms, improvements in psychosocial functioning, and good quality of life. Endorsements and specific guidelines have been published worldwide from leading governmental health agencies and international professional psychiatric organizations. These recommendations have been summarized so that practitioners can implement AE programs for individuals with SZ using group and/or individually tailored interventions to boost motivation and enhance self-efficacy for engaging in AE.

## Introduction

Schizophrenia affects approximately 1% of the global population and is for many individuals a severe and persisting mental disorder.^1^ Among the myriad of symptom presentations, Cognitive Impairment Associated with Schizophrenia (CIAS) is prominent and considered as a core feature of the disorder predating the classic positive symptoms often required in making a diagnosis.^2^ Importantly, these cognitive deficits often lead to profound functional disability and reduced quality of life for affected individuals. While antipsychotic medications aim to treat positive symptoms such as hallucinations and delusions, medications offer limited efficacy in addressing cognitive and ultimately functional deficits.^3^^,^^4^ Similarly, the evidence regarding effects of neurostimulation on CIAS is inconclusive.^5^ While the pro-cognitive effects of cognitive remediation have shown excellent promise,^6^^,^^7^ that does not preclude the development of additional non-pharmacological approaches.

Aerobic Exercise (AE) has had a substantial and beneficial impact on neurocognition for individuals with schizophrenia and related psychotic disorders as evidenced through multiple meta-analyses and comprehensive reviews. The evidence suggests that AE programs developed as adjunctive treatments can lead to significant cognitive gains for individuals with schizophrenia.^8–10^ These findings have important implications for the implementation of practical, non-pharmacological interventions to enhance cognitive functioning, reduce symptoms, and promote overall well-being in this population. Unfortunately, individuals with schizophrenia are known to lead sedentary lifestyles contributing additional risk for poor health, cognitive deficits, and poor functional outcomes.^11^^,^^12^ Consequently, there is a growing need and interest in non-pharmacological interventions prompting AE to emerge as a highly promising adjunctive therapy for enhancing cognitive functioning in schizophrenia.^13^

In this review, we examine the efficacy, mechanisms, rationale, and implementation guidelines surrounding the use of aerobic exercise in the treatment of cognitive dysfunction and related impairments associated with schizophrenia and psychotic disorders. We conducted a series of systematic searches of major literature databases, using the relevant keywords such “schizophrenia,” “Multi-Episode Psychosis” (MEP) or “First Episode Psychosis” (FEP), and then adding “aerobic exercise” and “cognition,” “physical health,” “social functioning,” “role functioning,” “psychiatric symptoms,” and “quality of life,” to identify a broad range of peer-reviewed articles pertaining to these topics. In addition, each of the study authors searched his/her own collection of published work. In doing so, we retrieved articles and aggregated the information that emerged from studies of MEP and FEP impacting different domains of functioning from Brain Regions to Quality of Life (QOL). We then created key summaries of the evidence, made recommendations for how practitioners could maximize the clinical benefits AE as a clinical and cognitive enhancement intervention in schizophrenia and related psychoses, and included the patient's perspective.

## Aerobic Exercise Positively Impacts Neurocognitive Functioning in Multi-Episode Schizophrenia

Numerous reviews and meta-analyses have demonstrated significant positive effects of AE on CIAS in Multi-episode Psychosis (MEP) over the past decade.^9^^,^^13–21^ Within this body of work, various studies have demonstrated the feasibility and sustained benefits from exercise interventions. In some cases the benefits lasted up to 6 months, with a few studies even indicating continued improvements in cognitive functioning over a year.^20^^,^^22–24^ These AE studies have admittedly varied in methodologies, sample size, effect size, and type of exercise intervention training. However, these studies have consistently demonstrated that global domains of neurocognitive functioning have been positively impacted by AE which have been linked to real world functioning:

**Attention** is a fundamental cognitive process essential for focusing on relevant environmental stimuli while ignoring distractions, which has been shown to improve following AE interventions.^13^^,^^14^ Research indicates that regular exercise enhances sustained attention, focused attention, and attentional control, thereby reducing attentional deficits characteristic of schizophrenia.

**Verbal Learning and Memory** improves with AE due to a positive effect on the ability to remember and recall information presented in a verbal form, such as words, sentences, stories, or any information conveyed through spoken or written language.^20^

**Working Memory** is the ability to temporarily hold and manipulate information for a few short moments during daily cognitive challenges which is essential for learning, reasoning, comprehension, and decision-making, is significantly and positively impacted by AE.^13^^,^^14^^,^^17^^,^^25^^,^^26^

**Processing Speed** has been shown to measurably improve in individuals with schizophrenia engaging in AE programs.^17^^,^^26^ Processing speed in particular has been shown to be associated with better functional outcomes in daily life.

**Executive functioning** is defined as a set of higher-order cognitive processes responsible for planning, problem-solving, and cognitive flexibility which also benefit from AE in schizophrenia.^18^^,^^20^^,^^26^ Individuals participating in AE interventions demonstrate improvements in inhibitory control, cognitive flexibility, and decision-making abilities. These enhancements facilitate adaptive functioning and may help mitigate executive dysfunction often associated with schizophrenia.

**Social cognition** is the study of how people process, store, and apply social information about people and social situations required to understand and navigate social interactions. Good social cognition is crucial for predicting behavior, forming relationships, and adapting to social environments. Fortunately, this skill has be shown to improve with AE, because good social cognation is fundamental for an individual’s successful social and role functioning.^27–29^

## Conclusion from Studies of Multi-Episode Psychosis

AE has been increasingly recognized as a valuable, non-pharmacological adjunctive therapy for individuals with schizophrenia and related psychotic disorders. There is a large, and rapidly growing body of research documenting the neurocognitive benefits of AE in several key domains that are robustly related to daily functioning. These findings support the inclusion of AE in therapeutic intervention programs to enhance cognitive health for individuals living with schizophrenia. The consistency of these findings across studies strongly supports the integration of structured and supervised AE programs into standard inpatient and outpatient treatment regimens.

## Aerobic Exercise Positively Impacts Neurocognitive Functioning in First Episode Psychosis

Several systematic reviews and meta-analyses of studies of individuals with FEP schizophrenia have shown there was a positive impact of physical activity(PA)-based interventions which mostly involved AE on cognitive functioning.^14^^,^^30–32^ The available evidence is very promising and suggests that AE and physical activity (PA) based interventions have small-to-large beneficial effects on the cognitive functioning of people with FEP, particularly on verbal and working memory, attention, processing speed, and social cognition. These findings are similar to the studies of MEP in that a higher dose of exercise, use of the group format, and the presence of social support, were all key factors associated with the effectiveness of exercise-based interventions. However, there seems to be consistent agreement that the maintenance of the cognitive effects, without continued intervention, does not usually endure overtime.

A review of individual studies of the effects of AE on cognitive functioning on FEP reveals that there was a mixture of sample sizes and research methodologies. This includes comparing AE to yoga^33^ and adding AE to cognitive training.^34–38^ Despite these variations in study methods, improvements favoring AE in key cognitive domains were found such as working memory, attention / concentration, and processing speed in comparison to control conditions. Although previous exercise studies in schizophrenia had experienced poor adherence, Firth et al^39^ found that young people in the early stages of illness were able to engage well with individualized exercise. Participants who were previously shown to be largely sedentary (at baseline) as assessed by the International Physical Activity Questionnaire (IPAQ), were in fact able to change their lifestyles. The participants achieved 107 min of moderate-to-vigorous activity per week for 10 weeks, thus surpassing the 90-min goal set by the research team. In that study, verbal short-term memory (STM) showed the largest pre-post changes, which exceeded practice effects. Furthermore, an increase in processing speed was positively associated with the amount of exercise attendance, indicating a dose–response relationship in FEP. Additionally, a 6-month follow-up of a sub-group of the sample indicated that the 55% who continued to exercise without structured exercise training support were able to maintain some of the cognitive gains achieved.^34^

In one multi-center, exercise intervention study by Hallgren et al,^35^ 91 outpatients with FEP (mean age = 30 years, 65% male) received usual care plus a 12-week supervised circuit-training program consisting of high-volume resistance exercises, aerobic training, and stretching. Participants exercised on average 13.5(SD = 11.7) times. Forty-eight percent completed 12 or more sessions. The primary study outcome was cognitive functioning assessed by the Cogstate Brief Battery and participation was associated with significant improvements among females for processing speed, visual learning, working memory, and visual attention (g = 0.43–0.69). A significant correlation was found between total training frequency and improvements in visual attention among males (r = 0.40, *p*<.05). The authors concluded that physical exercise is a feasible and safe adjunct treatment for FEP with measurable cognitive benefits.

## Summary and Conclusions from First Episode Studies

Reviews and meta-analyses in FEP generally support the notion that AE can have a positive impact on improving cognitive functioning in FEP, even though most effect sizes range from small to moderate.^30–32^ The evidence suggests that AE is a promising adjunctive treatment that could enhance the overall outcome in schizophrenia and related disorders when integrated into standard clinical programs. Despite these promising findings, the reviews also note some limitations such as heterogeneity of studies, variations in exercise protocols, duration, intensity, and participant characteristics. However, several strong and consistent findings have emerged all pointing to AE as a viable non-pharmacological, psychosocial treatment for the cognitive deficits in FEP.

Among several important points from FEP studies, we found that group-based interactive formats, professional guidance and structure, and support by peers have been suggested as factors that enhance the effectiveness of the impact of the AE interventions. Additional factors associated with intervention effectiveness were exercise dosage, e.g., intensity, duration of the session, and frequency. These factors provided some evidence of a dose response relationship between exercise and neurocognitive performance. Initially, researchers believed that AE interventions would have a greater impact in the early phase of illness. However, Fernandez-Abascal^32^ found that the effects on MEP individuals and FEP were mostly comparable and could not confirm a hypothesized heightened degree of responsiveness in FEP. Implications from these findings for practitioners are that both MEP and FEP are likely to benefit equally from AE. All of these important predictors of AE success and guidelines for interventions are expanded in the section (below) on **Implementation Guidelines for Practitioners**.

## Physical health of Individuals with Psychosis

The traditional treatment of a psychotic illness with pharmacological interventions has unfortunately been linked to increased physical health problems, such as cardiovascular disease and metabolic disorders.^12^ Aerobic Exercise (AE) helps mitigate the cardiovascular risks associated with antipsychotic medications and sedentary lifestyles common among individuals with schizophrenia.^40–42^

Regular aerobic activity has been shown to improve overall physical fitness, leading to increased energy levels and reduced fatigue. Regular AE has been found to enhance cardiovascular fitness, reduce obesity, and lower the risk of metabolic syndrome, which are common health concerns in individuals with psychosis. Improved physical health directly contributes to a better quality of life by increasing energy levels and reducing physical discomfort.^43^ Fernández-Abascal et al^32^ conducted a systematic review and meta-analysis on patients with FEP on inpatient and outpatient lifestyle interventions on diet and exercise effects on physical and psychological health. The interventions were generally well received, indicating good feasibility and acceptability within this population. The authors found that their interventions yielded significant positive effects on physical health (e.g., BMI, cardiovascular health) and psychological well-being (e.g., reduced symptom severity, improved mood) in patients with FEP. Enhanced physical health and cognitive functioning support greater independence and a higher quality of life.

## Activities of Daily Living in Individuals with Psychosis

While most Randomized Controlled Trials (RCTs) of AE in schizophrenia have focused on symptoms and cognitive outcomes, emerging evidence indicates that structured AE may improve functional capacity, global functioning, and disability outcomes that are closely related to the ability to perform Activities of Daily Living (ADLs).^44^ There is evidence indicating that ADLs can improve in people with schizophrenia, particularly when interventions directly target functional performance rather than symptoms alone.^45^ Also, there is some direct evidence that those with schizophrenia who engage in regular AE have demonstrated clinically meaningful improvements in ADLs, including personal care, household tasks, and meal preparation.^46^^,^^47^

The evidence suggests that AE improvements in cognitive functioning may in turn support more effective performance of everyday functional skills required for independent living. These improvements are mechanistically linked to improvements in everyday task performance and therefore represent plausible mechanisms through which AE may contribute to gains in ADLs. From a clinical perspective, these findings underscore the potential of AE as a valuable adjunctive intervention for addressing basic functional skills in schizophrenia, an area of persistent impairment that is often insufficiently improved by pharmacological treatment alone.

## Psychiatric Symptoms of Psychosis

### Mood Symptoms—Anxiety and Depression

Aerobic exercise (AE) has been shown to reduce a range of psychiatric symptoms in individuals with schizophrenia.^21^^,^^48^ In particular, AE has been associated with small-to-moderate, yet clinically meaningful reductions in depressive and anxiety symptoms, which may partially explain observed improvements in daily functioning.^49^^,^^50^ In addition, High-intensity Aerobic Training has demonstrated additional benefits, including improvements in maximal oxygen uptake (VO₂) alongside modest yet meaningful improvements in subjective well-being, with reductions in distress and state anxiety reported in patients with both depression and schizophrenia.^51^^,^^52^

Meta-analyses and reviews indicate that AE confers broad psychiatric benefits in schizophrenia, although effects on anxiety symptoms specifically remains less well characterized, while improvements on depressive symptoms are more consistent.^9^^,^^49^^,^^50^^,^^53^^,^^54^ Although individual studies have reported large effects of AE on depressive symptoms (e.g., Hedges’ g = −2.12,^53^ pooled estimates across studies suggest moderate overall effects (SMD ≈ −0.87) that did not reach statistical significance in some analyses only due to limited sample sizes and statistical power.^55^ Importantly, even modest reductions in depression and anxiety are clinically relevant in schizophrenia given the high prevalence of mood symptoms and their strong association with impaired functioning and reduced quality of life. Consistent with this, AE has been linked to improvements in health-related quality of life (HRQoL)^25^ and these effects appear to be partially mediated by reductions in depressive and anxiety symptoms.^49^

## Positive and Negative Symptoms

AE has been associated with a reduction in both positive symptoms, e.g., hallucinations, delusions and negative symptoms, e.g., lack of motivation, social withdrawal.^42^^,^^56^^,^^57^ This was found even when negative symptoms were compared to various types of control conditions.^44^^,^^54^^,^^55^^,^^58^^,^^59^ These findings are reviewed and summarized in two meta-analyses^57^^,^^60^ showing that AE was consistently superior to the provision of treatment as usual or occupational therapy. Subgroup analyses revealed that an AE treatment duration of two to three months, and an exercise duration of 100 to 220 min per week were most effective. In fact, recent estimates of the effects of AE suggest that approximately 50% of patients are showing clinically relevant improvement in symptom severity and 30% of patients experience significant improvement in daily functioning.^61^ Generally, the beneficial effects on positive and negative symptom severity were stronger in outpatients compared to inpatients and in general, the benefits were independent of the type of exercise performed. Negative symptom reductions are of particular importance because they are consistently correlated with improvements in daily functioning in schizophrenia.

## Aerobic Exercise and Real-World Functioning in Schizophrenia

Aerobic exercise (AE) has been increasingly examined for its potential psychosocial benefits, including improvements in real-world functioning among individuals with schizophrenia. A growing body of evidence indicates that individuals who are able to engage in AE or related forms of physical activity (PA) experience meaningful functional benefits. Exercise-based interventions have been associated with improvements in global functioning and social functioning across multiple studies.^13^^,^^29^^,^^62^ Consistent with these findings, positive associations have been reported between levels of physical activity and both social and occupational functioning.^63^

## Social Functioning

Social isolation and loneliness are risk factors for individuals with schizophrenia, with some studies identifying one or the other as linked to poor outcomes.^64^ Several AE studies in schizophrenia have suggested that group exercise activities provided behavioral training opportunities for social engagement and support from a physical activities coach. As hypothesized, those social skills are the type that enhance community integration. In addition, AE has been shown to improve social cognition, a related social functioning skill, in individuals with schizophrenia^9^^,^^29^ as was confirmed in meta-analyses and reviews.^27^ These types of exercise interventions have been used successfully to increase social connections, and reduce social isolation and loneliness in this population which is very important because these are risk factors for poor outcomes in schizophrenia.^29^^,^^65–67^

## School and Work Functioning

Currently, there is limited direct evidence connecting AE to better academic performance in school for individuals with schizophrenia. However, several studies highlight the broader benefits of AE on cognitive functioning, which are indirectly associated with improved academic performance. Although not directly studied in a sequencing analysis, these improvements are linked to cognitive domains such as attention and processing speed which have been found to be relevant for school functioning.^68^ This supports the notion that AE interventions have been linked to increased productivity and better work performance as well in individuals with schizophrenia.^49^^,^^69^ Again, although there might not be direct connections, AE has been found to improve cognitive functioning in FEP and MEP individuals with schizophrenia which can enhance academic, role, and work performance.^36^

## Quality of Life

The evidence suggests that AE offers multiple benefits that collectively enhance the Quality of Life (QOL) for individuals with schizophrenia. One comprehensive review found that supervised AE has been consistently shown to improve QOL in schizophrenia patients with benefits for physical and mental health, cognition, and overall functioning.^70^ Research shows that engaging in physical activity improves Health-Related Quality of Life (HRQoL) and activities of daily living in schizophrenia patients.^25^^,^^71^ In fact, exercise and movement has been found to promote an overall sense of well-being.^72^ Regarding possible biological mechanisms, AE promotes the release of endorphins and other neurotransmitters that enhance mood and overall well-being and helps regulate sleep patterns, which are crucial for optimal mental health and good daily functioning.^73^ This form of enhanced functional outcome supports a higher level of independence and a more fulfilling QOL in individuals with living with schizophrenia.

## Neurobiological and Brain Mechanisms

Evidence suggests that engaging in regular AE initiates a number of neurobiological changes that enhance the structure and function of several brain regions and networks involving prefrontal cortex and hippocampal areas. These structural and functional adaptations are assumed to correspond to enhanced cognitive performance, as shown for hippocampal neuroplasticity in response to AE in people with schizophrenia.^74–77^ Engaging in regular AE, e.g., walking, running, cycling, and swimming, has been proposed to stimulate neurogenesis, synaptogenesis, and angiogenesis, particularly in brain regions such as the prefrontal cortex and hippocampus.^14^ Functional neuroimaging studies have revealed enhanced connectivity within prefrontal and hippocampal circuits following AE interventions.^78^ In this regard, the hippocampus^78^ as part of the default-mode network,^79^ the prefrontal cortex,^80^^,^^81^ the cortico-striato-pallido-thalamo-cortical loop or the cerebello-thalamo-cortical pathway^79^ reflect promising neural targets for AE interventions. The increase in hippocampal volume is important because that brain region is critical for learning and memory processes.^34^^,^^78^^,^^82^ The theory and understanding of these neurobiological effects of AE in individuals with schizophrenia has been a driving force for the development of physical exercise rehabilitation programs and interventions.

## Neurobiological Changes following Aerobic Exercise and their Cognitive Implications

The cognitive benefits of AE in schizophrenia are supported by neurobiological mechanisms involving these structural and functional changes in the brain that are associated with improvement in cognitive functioning.^83^^,^^84^ Hypothesized mechanisms include the fact that exercise increases blood flow and oxygenation to brain areas.^14^^,^^75^^,^^85–87^ Moreover, exercise promotes neuroplasticity in schizophrenia, facilitating the formation of new neural connections and synaptic remodeling within cognitive networks in the prefrontal cortex.^81^^,^^88^ In fact, once activated, AE can alter certain brain mechanisms that increase Brain-Derived Neurotrophic Factor (BDNF) or reduce Interlukin-6 (IL-6) which can enhance cognitive function, reduce negative symptoms, and symptoms of depression and anxiety.^36^^,^^59^^,^^81^^,^^87^^,^^89–91^ These brain changes could theoretically lead to improvements in several key cognitive domains, which in turn enhance daily functioning and overall quality of life.

## Implications of the Positive Impact of Aerobic Exercise on the Brain and Neurocognition

We recommend that the practitioner consider using brain plasticity science in providing the optimal rationale for exercise interventions. Understanding their nature will help provide a justification for supporting the participation of an individual with schizophrenia in AE intervention programs. As part of this explanation, individuals need to know that these brain mechanisms have “plasticity” and are modifiable through AE. Given the current state of evidence linking brain change to cognitive improvements, further large-scale AE studies are required to improve our understanding of neurobiological mechanisms that drive cognitive improvements in schizophrenia. However, there is sufficient evidence to move forward currently with AE interventions to support pro-neuronal development and neurogenesis in key brain areas that are associated with cognitive functioning.

## Implementation Guidelines for Practitioners

Aerobic exercise (AE) and physical activity (PA) have clearly been established as a beneficial adjunctive, non-pharmacological interventions to aid with illness management for individuals living with schizophrenia and related psychotic disorders.^92^ Specific implementation guidelines are available for clinicians and practitioners for how to thoughtfully prescribe AE within an appropriately informed and supportive framework (Table 1).^93^^,^^94^ The first step is to have a thoughtful and informed conversation with a patient about AE as a treatment strategy. The overall framework is to employ a psychoeducational and goal oriented approach as a mental health professional would with any intervention used to facilitate recovery from schizophrenia. In terms of the specific AE exercise types, interventions such as brisk walking, jogging, cycling, and swimming have all been successfully implemented in individuals with schizophrenia and related conditions. Tailoring exercise programs to meet the individual needs and preferences can significantly improve both adherence and overall effectiveness. Interventions that implement a sufficient dose of exercise, in supervised group settings or individually are not only feasible but can be highly effective. One option is that AE has been shown to be effective when provided as part of an interdisciplinary Team.^95^

**Table 1:** Summary of Recommendations for Practitioners: Implementing Aerobic Exercise Programs for Individuals with Schizophrenia and Related Psychotic Disorders

1) Identify facilities and providers in the community as resources such as local YMCA or YWCA, health care facilities, mental health outpatient programs, local gyms, research studies, or private practitioners that offer aerobic exercise activities or structured programs.
2) Be prepared to promote supervised group and/or individual aerobic exercise programs by developing a set of collaborations with an agency or individual providers in the community as they can reliably ensure adherence to exercise programs and improve clinical outcomes.
3) Promote structured formats whenever possible since this format enhances motivation and social support; combine group with individual-participant based interventions whenever possible for management of mood or psychotic symptom profiles or for individuals with low fitness levels.
4) Follow evidence-based dosing by aiming for 150 minutes per week over 2–3 sessions per week, 45–60 minutes per session of at least moderate intensity exercise, sustained for a minimum of 12–24 weeks. Then evaluate progress and reset goals!
5) Offer simple, accessible activities such as brisk walking, cycling, jogging, or swimming, and adapt those formats to group or individual-based preferences to enhance engagement.
6) Actively enhance motivation and overcome negative attitudes through psychoeducation, goal setting by focusing on small steps to reinforce success by providing immediate feedback, and using social support mechanisms such as peer group support, cell phone technology, family support, or a buddy-system.
7) Promoting aerobic exercise programs often entails helping individuals overcome cognitive challenges by providing the cognitive “scaffolding” needed to overcome basic logistics such as resource identification, time management, scheduling, planning, anticipating transportation needs, and building-in appointment reminders.
8) The practitioner can increase the likelihood of success by tailoring programs to the individual participant’s physical activity capacity, symptom fluctuations and management needs, and logistical limitations to maximize adherence and functional gains.

Overall, several important issues need to be considered by the practitioner when prescribing AE or physical activity as a treatment intervention in schizophrenia: 1) Optimal timing and dose of the AE intervention for maximum benefits, 2) Benefits of group aerobic exercise training, 3) Role of motivation for exercise participation and adherence, and 4) An individual’s perspective on the impact of AE on cognition, health, symptoms, functioning, and QOL. These options, treatment settings, intervention approaches, and the challenges they pose will be addressed in the sections that follow.

## Optimal Timing and Dose of Intervention for Maximum Benefits in Schizophrenia

Evidence suggests that adherence is maximized when programs emphasize active participation over time with a mindset toward long-term sustainability rather than short-term gains. A weekly exercise dose of 150 minutes of moderate aerobic activity over 2-3 sessions of 45-60 minute duration per week with an active warm-up and cool-down is consistent with the American College of Sports Medicine and U.S. Department of Health & Human Services Guidelines.^96^ These are consist with AE effectiveness duration and frequency recommended by the European Psychiatric Association (EPA) guidelines of 2–3 supervised sessions per week, lasting 45–60 minutes of at least moderate intensity,^21^ typically defined as 60-70% of maximum heart rate. More recent findings suggest that high-intensity exercise (70-80% maximum heart rate) may yield even more substantial improvements, particularly in cognitive function, which is closely tied to overall daily functioning.^26^^,^^36^ Interestingly, more frequent exercise sessions appear to be particularly effective in improving psychosocial functioning.^60^ In general, higher levels of adherence to AE programs are generally associated with increasing benefits for cognitive domains, negative symptoms, interpersonal relations, and functional outcome for individuals with schizophrenia. Regarding the total duration over time, existing studies consistently indicate that interventions of 12–24 weeks produce the most robust effects.^13^^,^^36^^,^^95^^,^^97^^,^^98^

## Benefits of Group Aerobic Exercise Training for Schizophrenia

**Advantages:** There is evidence suggesting that AE in group settings has some advantages compared to exercise in individual settings. Supervised group exercise is associated with higher attendance and retention compared to unsupervised individual exercise. In fact, exercise in supervised groups facilitated by professional trainers resulted in higher attendance and retention than *un*supervised or solitary exercise.^9^^,^^13^^,^^14^^,^^31^ Enhancements in global cognition and in several cognitive domains appears to be particularly supported by group-based interventions.^13^^,^^31^ Moreover, group-based interventions foster a sense of social support and a spirit of camaraderie. Participating in group exercise activities provides opportunities for social interaction that can reduce feelings of isolation. In addition, group training has an advantage in improving cognitive function particularly in social cognition and cognitive flexibility. For individuals with schizophrenia, who often experience social isolation,^99^ these sessions can provide valuable opportunities to engage socially with others, develop social skills that are critical for everyday functioning.^100^ Furthermore, group dynamics can enhance engagement and accountability making adherence with exercise routines more likely. The social support and sense of accountability found in group settings, such as through goal setting in a “Bridging Group” often leads to better adherence to the exercise program, a critical factor in ensuring long-term benefits.^36^^,^^101^

**Disadvantages:** However, disadvantages of the group format could include that they sometimes result in distractions for some participants that could reduce exercise intensity. In addition, at a group level, exercise induced negative effects may occur among individuals with schizophrenia. This could be because the need to accommodate multiple group participants means the exercises may be less personalized to individual needs.^61^ Also, adherence and fitness improvements may not necessarily translate directly into symptom benefits without that individualized, personal attention sometimes needed for those with schizophrenia.

## Benefits of Individual Aerobic Exercise Training for Schizophrenia

**Advantages:** Individual training allows for tailoring to physical abilities, fitness levels, and symptom profiles. This approach often enables higher exercise intensity and potentially greater improvements in physical fitness and symptom management especially for those starting at lower levels of fitness. Regarding symptom reduction, individual training may have a slight advantage, as personalized attention often leads to greater improvements in managing symptoms like anxiety, depression, and hallucinations. Also, individual session flexibility in scheduling may support adherence.

**Disadvantages:** Group training may be more effective for social cognition and sense of commitment adherence. Also, individual sessions are often less accessible and more costly. The lack of peer social support can reduce motivation.

## Summary on Group vs Individual Approach to Aerobic Exercise Programs

While both group and individual AE training programs have benefits for schizophrenia patients, the optimal format may depend on individual preferences and needs. Also, both group and individual training formats have been shown to improve symptoms and cognition with slight edge in favor of group training for FEP.^31^ A combined approach, incorporating the social benefits of group training with the tailored focus of individual sessions may offer the most comprehensive benefits (whenever feasible). Apart from that, professional supervision with a certified trainer seems to be a key factor for maximum clinical and neurocognitive benefits.^13^^,^^20^^,^^60^

## The Role of Motivation for Exercise in Schizophrenia

Despite robust evidence demonstrating the physical, cognitive, and psychological benefits of exercise for those living with schizophrenia, many individuals experience substantial difficulty initiating and/or maintaining regular physical activity.^102^^,^^103^ Prominent negative symptoms, such as apathy, diminished initiative, and social withdrawal combined with impairments in executive functioning and planning, can create significant motivational barriers that limit sustained engagement.^94^^,^^104^ Accordingly, motivation is often widely recognized, by individuals with schizophrenia and professionals alike, as a central barrier of successful participation in exercise-based interventions. Both intrinsic motivation (e.g., internal desire stemming from a sense of personal satisfaction) and extrinsic motivation (e.g., structured routines, professional coaching, reminders, and social accountability) can influence poor or good adherence to exercise programs.

A range of strategies used in clinical trials has been shown to strengthen motivation. Psychoeducation enhances a participant’s understanding of the physical and mental health benefits of activity, and increasing willingness to engage.^105^ Goal setting and graded task progression allow individuals to experience early mastery, which increases self-efficacy and encourages continued participation. Motivational enhancement strategies, including supportive feedback, motivational interviewing, Cognitive Behavioral Therapy, and structured reinforcement have also demonstrated positive effects on adherence.^32^ Social support mechanisms, such as peer-led sessions, peer facilitated, or trainer-facilitated group exercise, further enhance adherence by fostering a sense of community, accountability, and positive social engagement.^25^^,^^106^ Integrating these motivational components into exercise interventions and tailoring them to an individual’s symptom profile, cognitive style, and social needs can substantially mitigate common barriers to participation thereby improving long-term engagement.^107^

## The Role of Self-Efficacy

A growing body of research highlights self-efficacy, an individual’s belief in his/her ability to successfully engage in exercise as a key psychological mechanism influencing motivation and adherence in schizophrenia (Table 2).^29^^,^^42^^,^^108^^,^^109^ Individuals with higher exercise-related self-efficacy are more likely to initiate physical activity, persist through challenges, and transition from contemplation to action or maintenance stages of behavior change.^105^^,^^110^ Although much of the evidence is correlational, these findings suggest that strengthening perceived competence may help counteract the lack of motivation linked to negative symptoms. Even though research directly linking self-efficacy to functional outcomes remains limited, preliminary evidence suggests that improved motivation, competence, and activity engagement may indirectly contribute to better functioning by increasing behavioral activation, promoting social contact, and enhancing mood regulation.^14^^,^^21^

**Table 2:** Several Psychosocial Intervention Components for Aerobic Exercise Appear to Enhance Self-efficacy and Promote Behavioral Activation That Can Add Benefits for Individuals with Schizophrenia and Related Psychotic Disorders

1) Structured, predictable exercise routines which provide external scaffolding for initiation and implementation of an exercise program for group or individual participation that help reduce the cognitive burden for participants that require advance planning will increase the likelihood of success for a positive outcome.
2) Trainer-guided group participation and skill acquisition which allows participants to learn aerobic exercise techniques, experience mastery, and build self-efficacy and confidence in their abilities, are factors that are likely to generalize to other daily functional and social domains such as with family and friends or in related school or work settings.
3) Incremental aerobic exercise goal achievement, within a goal setting framework that reinforces success and through a steady pattern of engagement and small rewards that are consistent with behavioral activation frameworks and contrary to sedentary behavior can be used as an intervention to reduce depression and negative symptoms.
4) Positive social interactions that are experienced in supervised group aerobic exercise formats, can reduce feelings of isolation and increase enjoyment in social interaction and promote perceived social reward systems that create low-pressure opportunities to practice pro-social behaviors in a safe environment that reduces anxiety and dysphoria.

Taken together, these findings indicate that interventions targeting self-efficacy, behavioral activation, providing structure and support, and goal setting will help overcome motivational barriers and support durable engagement in exercise among individuals with schizophrenia.^102^^,^^110^^,^^111^ The use of AE programs can have psychological benefits as well in that exercise can boost self-esteem and self-efficacy, leading to greater confidence in managing daily activities and social relationships.

## Patients’ Perspectives on Aerobic Exercise to Improve Cognition and Health

Overall, individuals with lived experience of psychosis or schizophrenia often describe AE as having meaningful and broadly positive effects on their physical well-being, including cognition, social cognition, daily functioning, and QOL (Table 3). Specifically, individuals describe aerobic exercise as helping them “think more clearly,” stay focused for longer periods, and feel more mentally organized, reporting perceived benefits for attention, working memory, and processing speed.^39^^,^^105^ Some individuals report that regular exercise enhances motivation and “mental energy,” which they viewed as essential for engaging in cognitively demanding tasks. Group-based AE reduces personal isolation and was seen as beneficial for social cognitive functioning.^106^^,^^112^ Participants noted that exercising with others provides structured opportunities for social interaction, reducing feelings of social isolation, and increasing social confidence. Many also emphasize the broader health benefits of exercise, including weight control, improved sleep, reduced medication side effects, improving mood, and greater day-to-day physical vitality.^21^^,^^90^

**Table 3:** Schizophrenia Patients With Lived Experience: Perspectives on Perceived Benefits, Barriers, and Facilitators That Can Function as an Add in Boosting Aerobic Exercise Participation

**Domain**	**Key Themes**	**Examples of Patient Perspectives**	**Representative Citations**
Perceived Benefits	Cognitive functioning	Improved concentration, clearer thinking, better memory, less “mental fog”	Soundy et al., 2014; Firth et al., 2016
Perceived Benefits	Social cognition	Increased confidence interacting with others, improved social skills through group interaction	Soundy et al., 2015
Perceived Benefits	Emotional well-being	Reduced stress, improved mood, increased vitality	Rosenbaum et al., 2015
Perceived Benefits	Physical health	Weight management, better sleep, more energy, improved cardiovascular fitness	Stubbs et al., 2018
Perceived Benefits	Empowerment and identity	Feeling capable, gaining a sense of control, engaging in meaningful activity	Soundy et al., 2014
Barriers	Negative symptoms	Low motivation, apathy, reduced initiative, difficulty starting activities	Firth et al., 2016; Vancampfort et al., 2012
Barriers	Cognitive impairments	Challenges with planning, organization, sustaining attention; forgetting routines	Firth et al., 2016
Barriers	Physical limitations	Low baseline fitness, fatigue, medication-related weight gain	Stubbs et al., 2018
Barriers	Psychological barriers	Fear of stigma, low confidence, anxiety in social or group settings	Soundy et al., 2014; Soundy et al., 2015
Barriers	Environmental barriers	Limited access to safe environments, transportation difficulties, unpredictable routines	Vancampfort et al., 2018
Facilitators	Structured routines	Predictable schedules, step-by-step guidance, clear expectations	Vancampfort et al., 2012
Facilitators	Trainer or coach support	Encouragement, modeling exercises, positive feedback, sense of safety	Soundy et al., 2015; Firth et al., 2016
Facilitators	Group formats	Sense of belonging, accountability, shared goals, reduced isolation	Soundy et al., 2014; Vancampfort et al., 2018
Facilitators	Goal setting and mastery	Experiencing success, tracking progress, building self-efficacy	Firth et al., 2016
Facilitators	Tailored programs	Pace matched to ability, adaptations for symptoms, preference-based options	Stubbs et al., 2018

**Note:** Full references can be found in the text in the section that discusses patient perspectives titled: **Patients’ Perspectives on Aerobic Exercise to Improve Cognition and Health**

Despite these positive perceptions, individuals also identify several barriers that interfere with consistent participation in AE activities. Individuals are aware of issues such as lack of motivation, low initiative, and reduced pleasure can make starting or maintaining exercise routines difficult. Cognitive impairments, particularly difficulties with planning, organization, and sustaining attention, can further complicate adherence to exercise programs.^39^ Additional barriers include low fitness levels, fear of stigma, and limited access to safe environments or structured programs. At the same time, individuals highlight important facilitators: clear instructions, predictable routines, supportive trainers, group formats that foster accountability, and environments that feel safe and nonjudgmental.^103^^,^^105^^,^^106^ Many individuals express a preference for supervised or guided sessions, noting that these approaches increase motivation, build confidence, and help them experience early success. All of these are factors that reinforce continued participation.

The perspectives of those individuals with lived experience clearly guide us in the same direction as empirical researchers have from years of research with several important implications for clinical implementation. First, exercise interventions should incorporate structured, predictable, and supervised formats, as these features closely match patient preferences and address motivational and cognitive challenges. Second, group-based or peer-supported interventions may enhance both adherence and social cognitive outcomes. Third, programs should initially include initial low-intensity activities, graded goal progression, and opportunities for early mastery to build self-efficacy, a known predictor of sustained engagement. Finally, incorporating patient-centered feedback mechanisms throughout the intervention can help tailor programming to individual needs and maximize perceived cognitive and health benefits. By integrating these perspectives into an implementation plan, interventions can more effectively support engagement and actualize the full therapeutic potential of AE for cognitive, functional, health, QOL, and psychosocial outcomes in schizophrenia.

## Overall Summary and Conclusions

Overall, aerobic exercise (AE) has emerged as a potentially robust, highly promising, non-pharmacological, adjunctive therapy for improving cognition in individuals with schizophrenia and related disorders. Demonstrated improvements have been found across key cognitive domains such as attention, memory, processing speed, executive function, and social cognition. These improvements are linked directly to functional gains and enhancing quality of life. Preliminary evidence points to structural and functional brain adaptations through plasticity driven mechanisms, such as increased hippocampal and frontal volume and enhanced neural connectivity, suggesting neurobiological mechanisms underlying these beneficial effects that have been observed. Regarding implementation in clinical practice, no additional evidence is needed. Aerobic exercise has been recommended world-wide for adoption as a best practice to improve a wide spectrum of recovery outcomes in schizophrenia through reports and guidelines from the World Health Organization,^107^ the European Psychiatric Association,^21^ the Lancet Psychiatry Physical Health Commission,^92^ and other major health/scientific groups.^113–115^ Future studies should aim to establish the extent to which specific and specialized interventional approaches can positively impact patient specific outcomes, or alternatively if participant preferences are the determining factor in “what works best” for AE adherence and efficacy. Schizophrenia's complex etiology means that AE cannot be the perfect intervention that addresses all treatment indications. However, AE’s numerous benefits underscore the importance of incorporating structured physical exercise into a comprehensive and multidisciplinary treatment program. Ultimately, AE represents a feasible and effective strategy to enhance cognitive function, improve mood and reduce psychotic symptoms, promote health, and significantly improve the quality of life for individuals living with schizophrenia and related psychotic disorders.
